# Morphological characteristics of *Candida albicans, Candida krusei, Candida guilliermondii*, and *Candida glabrata* biofilms, and response to farnesol

**DOI:** 10.14202/vetworld.2021.1608-1614

**Published:** 2021-06-22

**Authors:** Nadezhda Sachivkina, Irina Podoprigora, Dmitry Bokov

**Affiliations:** 1 Department of Microbiology and Virology, Medical Institute, Peoples’ Friendship University of Russia, Moscow, Russia; 2 Institute of Pharmacy, Sechenov First Moscow State Medical University, Moscow, Russia

**Keywords:** *Candida albicans*, *Candida glabrata* biofilms, *Candida guilliermondii*, *Candida krusei*, farnesol, optical density, quorum sensing

## Abstract

**Background and Aim:**

Different *Candida* species isolated in humans and animals have different types of parasite activity. The most pathogenic species is *Candida albicans* followed by *Candida tropicalis*. However, the effects of the morphology of *Candida krusei, Candida guilliermondii*, and *Candida glabrata* biofilms on the pathogenicity of these species have not been fully characterized. To the best of our knowledge, there is no literature on the effect of farnesol on rare *Candida* species. This study aimed to check the effect of different farnesol concentrations on the species *C. krusei, C. guilliermondii*, and *C. glabrata* compared with the strain *C. albicans* ATCC 10231, which has been widely studied, and is a strong producer of biofilms.

**Materials and Methods:**

We studied the morphological and densitometric parameters of biofilms produced by *Candida* species under the influence of the drug farnesol (Sigma-Aldrich, St. Louis, MO). We used a heart brain broth with the addition of 2% bovine blood serum in 96-well plates. To each well, we added 100 mL of *C. albicans, C. krusei, C. guilliermondii*, or *C. glabrata* culture, and 0.2-400 mM farnesol. The microliter plates were cultured with the lid closed at 37°C for 48 h. Then, the liquid was removed, and the wells were washed 3 times with 200 mL phosphate buffer solution (pH 7.3). Biofilm fixation was performed using 150 mL of 96% ethanol for 15 min. Then, the microliter plates were dried for 20 min at 37°C, a 0.5% solution of crystalline violet was added, and the plates were placed in an incubator at 37°C. After 5 min, the contents of the wells were removed, washed 3 times with 200 mL of phosphate buffer solution (pH 7.2), and dried. The dye was extracted by washing with 200 mL of 96% ethanol for 30 min. The results were obtained using a photometric analyzer of enzyme immunoassay reactions at an optical density (OD) wavelength of 450 nm.

**Results:**

All of *Candida* spp. strains tested were susceptible to farnesol at concentrations ranging from 0.8 to 400 mM for *C. albicans, C. krusei*, and *C. guilliermondii*, and 12.5 to 400 mM for *C. glabrata*.

**Conclusion:**

This study provides new insights into the use of farnesol against biofilms produced by *Candida* species, but further studies *in vivo* are necessary to evaluate the effectiveness of the reduction of OD. To the best of our knowledge, the antimicrobial activity of farnesol against *C. krusei, C. guilliermondii*, and *C. glabrata* has not been reported previously, although studies have confirmed the inhibitory effect of farnesol on the growth of different microorganisms.

## Introduction

The last decade has been characterized by a gradual revision of our understanding of microorganisms as single-celled individuals. Evidence is accumulating to support the contention that they are integral “superorganisms,” leading a social way of life. Biofilms are a key factor that ensures the preservation of many species [1]. In the external environment, about 99.9% of all microorganisms are able to form biofilms. Biofilms are unique formations consisting of living cells immersed, in the form of microcolonies, in an exopolymer, a polysaccharide matrix which accounts for about 85% of the volume of the biofilm [2]. The matrix produced by cells in biofilms provides physical protection of the cells from immune system factors such as antibodies and macrophages, and from bacteriophages, and hinders the penetration of antibiotics, contributing to antibiotic resistance. Both bacteria and fungi form biofilms. Among the fungi, *Candida* spp. and, particularly *Candida albicans*, are important in human pathology, due to their extremely high resistance to antimycotics. To suppress the metabolic activity of *C. albicans* biofilms, several times higher concentrations of amphotericin B, fluconazole, itraconazole, and ketoconazole are required than for yeast planktonic cells [3,4]. It is, therefore, important to develop new approaches to the treatment of infections accompanied by the formation of biofilms.

The first data on signaling molecules in fungi were obtained a quarter of a century ago and have been summarized in a number of fundamental reviews [4-6]. Recent studies carried out using ­molecular biology and biochemistry approaches have significantly expanded the known range of fungal signaling molecules and the chemosignaling systems that recognize them. These studies indicate a high prevalence of intra- and inter-specific chemocommunication (quorum sensing [QS]) in the fungi, indicating microorganism universality in the living world. Several decades ago, sesquiterpene E, E-Farnesol, and the related farnesylic acid were identified in *C. albicans*. These compounds are produced in response to an increase in the density of fungal cell culture. They block the formation of hyphae and prevent the formation of surface biofilms, leading to a decrease in the pathogenicity of *C. albicans*. Farnesylic acid functions as a QS molecule in only one strain of the fungi *C. albicans*, ATCC 10231, while farnesol is an active regulator of hyphal formation in most other strains of *C. albicans*, as well as in the related fungi *C. albicans*, *Candida tropicalis*, and *C. dubliniensis*. In the fungus *C. parapsilosis*, farnesol suppresses the formation of surface biofilms, but does not affect the process of hyphae formation [5-7]. The mechanism of destruction of the surface biofilms formed by *C. albicans* and related fungi is based on the achievement of a high enough concentration of farnesol to regulate the expression of genes responsible for the synthesis of the proteins that determine the structural organization of surface biofilms.

There has been considerable research into the effects of farnesol on *C. albicans* biofilms, including some of our previous work. However, the biofilms of rare species, such as *Candida krusei*, *Candida guilliermondii*, and *Candida glabrata*, remain unexplored. *C. albicans* is a diploid yeast-like fungus. *C. krusei* and *C. guilliermondii* are also diploid, while *C. glabrata* is a haploid microorganism and is not able to form mycelial structures.

This study aimed to check the effect of different farnesol concentrations on the species *C. krusei, C. guilliermondii*, an*d C. glabrat*a compared with the strain *C. albicans* ATCC 10231, which has been widely studied, and is a strong producer of biofilms.

## Materials and Methods

### Ethical approval

This work with the microorganisms does not require authorization from the Ethics Committee.

### Study period and location

The study was conducted from January to March 2021. The samples were processed at the Department of Microbiology and Virology, Medical Institute, Peoples’ Friendship University of Russia, Moscow, Russia.

### Strains

The study of biofilms and phenotypic features was carried out using the reference strain of *C. albicans* ATCC 10231 https://vkpm.genetika.ru/katalog-mikroorganizmov/cat30012009367/.

*C. krusei, C. guilliermondii*, and *C. glabrata* strains were obtained from Veterinary Clinical Materials (Supplementary data can be available from the corresponding author). The microorganisms were identified using the matrix-activated laser desorption/ionization technology Bruker Daltonik MALDI Biotyper (Bruker Daltonik Inc., Billerica, MA, USA). After taking into account, the values of the X score, which ranged from 0 to 3, values from 2 to 3 were considered successful. A result with a score of more than 2.3 was considered to be highly reliable. *C. krusei* had a score value of 2.430, *C. guilliermondii* had a score value of 2.255, and *C. glabrata* had a score value of 2.407.

### Culture media and reagents

The microorganisms were cultured for 24 h at 37°C using the following media: Heart brain broth (HiMedia, India), Sabouraud agar (BioMerieux, France), and bovine blood serum (FSUE NPO “Microgen” of the Ministry of Health, Russia). The study of morphological and densitometric parameters of biofilms was performed using the drug farnesol (“Farnesol,” “Sigma–Aldrich,” Germany).

### Morphological characteristics of biofilms of different types of *Candida*

The preparations were taken in a loop from the control wells (one column), the drop was placed on a slide, not fixed, covered with a cover glass, not stained, and examined under oil immersion using an optical microscope (BIOMED MS-1 Stereo [BIOMED, Russia]).

### The effect of farnesol on the formation of *Candida* biofilms

In the experiment, we used a heart brain broth with the addition of 2% bovine blood serum (HBBr+S). Using the serum, we aimed to create conditions as close as possible to the *in vivo* situation. In the presence of serum, the formation of hyphae is stimulated, and hyphae formation is the most important criterion for the pathogenicity of *Candida* in humans and animals [8,9].

An automatic pipette was inserted into the wells of a 96-well plate (Medpolymer, St. Petersburg, Russia):

- 100 μL HBBr+S in each of the 12 holes of the first row A.
- 100 μL of farnesol was added at an initial concentration of 400 μM to the second well of the first row. The first hole was left as a control. In the second hole, the volume was 200 μL, and the concentration of farnesol was 200 μM. By successive transfer of 100 μL of the solution from the second well to the third, from the third to the fourth…, etc., we reduced the concentration of farnesol by half each time.
- Then, in each well of the first row, starting from the first, we added 100 μL of *C. albicans* culture ATCC 10231 in HBBr+S at a concentration of 4 units (McFarland).
- *C. krusei*: Second row;
- *C. guilliermondii*: Third row;
- *C. glabrata*: Fourth row.

An overview of the sequence of stages of the study into the effect of farnesol on the formation of *Candida* biofilms is presented in Table-1.

**Table-1:** Stages of the study of the farnesol effect on the biofilm formation.

	1	2	3	4	5	6	7	8	9	10	11	12
Action 1	HBBr+S 100 µL	HBBr+S 100 µL	HBBr+S 100 µL	HBBr+S 100 µL	HBBr+S 100 µL	HBBr+S 100 µL	HBBr+S 100 µL	HBBr+S 100 µL	HBBr+S 100 µL	HBBr+S 100 µL	HBBr+S 100 µL	HBBr+S 100 µL
Action 2		+Farnesol										
Action 3	Not titrated	Transfer 100 µL	Transfer 100 µL	Transfer 100 µL	Transfer 100 µL	Transfer 100 µL	Transfer 100 µL	Transfer 100 µL	Transfer 100 µL	Transfer 100 µL	Transfer 100 µL	Transfer 100 µL
The concentration of farnesol	Control – no farnesol	200 μM	100 μM	50 μM	25 μM	12.5 μM	6.3 μM	3.1 μM	1.6 μM	0.8 μM	0.4 μM	0.2 μM
Action 4	+100 µL of culture	+100 µL of culture	+100 µL of culture	+100 µL of culture	+100 µL of culture	+100 µL of culture	+100 µL of culture	+100 µL of culture	+100 µL of culture	+100 µL of culture	+100 µL of culture	+100 µL of culture
Action 5	We are waiting for 48 h											

The total volume of the wells was 200 μL. The experiment was repeated 3 times, 3 μL plates were used, and four rows were used per plate. The microliter plates were cultured with the lid closed at 37°C for 48 h. Then, the liquid was removed, the wells were washed 3 times with 200 μL phosphate buffer solution (pH 7.3). Biofilm fixation was performed with 150 μL of 96% ethanol for 15 min. Then, the microliter plates were dried for 20 min at 37°C, a 0.5% solution of crystalline violet was added, and the plates were placed in an incubator at 37°C. After 5 min, the contents of the wells were removed, washed 3 times with 200 μL of phosphate buffer solution (pH 7.2), and dried. The dye was eluted using 200 μL of 96% ethanol for 30 min. The results were obtained using a photometric analyzer of enzyme immunoassay reactions AIFR-01 UNIPLAN (Picon, Russia) at an optical density (OD) wavelength of 450 nm [10,11].

### Statistical analysis

The experimental data were processed using descriptive and inferential statistics. Means and standard deviations of the OD and adhesive properties were calculated using Microsoft Excel. The differences between the means of samples and that of the control were determined using Student’s t-tests, and statistical significance of the differences was set at p≤0.05.

## Results

### Morphological characteristics of different types of *Candida* biofilms

**Figure-1:**
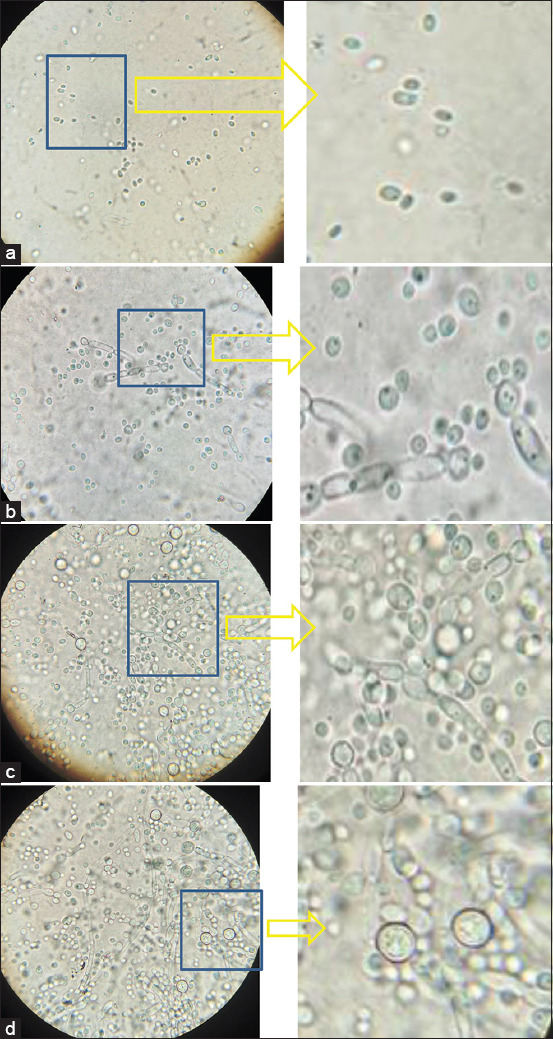
Morphology of *Candida albicans* biofilm ATCC 10231, 10×100, immersion. (a) 6 h of incubation; (b) 24 h of incubation; (c) 36 h of incubation; (d) 48 h of incubation.

*C. albicans* (Figure-1) can be differentiated from other members of this genus by the presence of chlamydospores and pseudomycelia. Chlamydospores are large formations at the ends of hyphae, most often round in shape with a thick wall, with a diameter of 7-13 m. It is not difficult to assess their presence; they are usually clearly visible without any staining.

**Figure-2:**
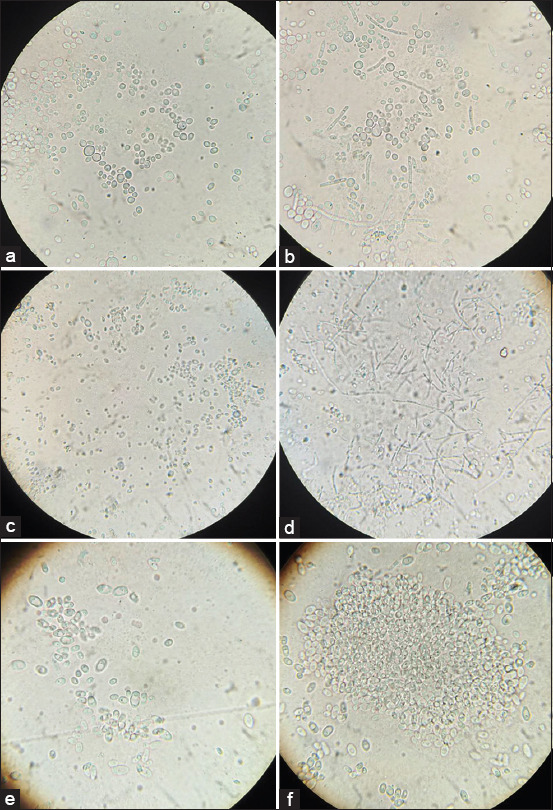
Morphology of yeast-like fungi of the genus *Candida*, 10×100, immersion. (a) *Candida krusei* 24 h of incubation; (b) *C. krusei* 48 h of incubation; (c) *Candida guilliermondii* 24 h of incubation; (d) *C. guilliermondii* 48 h of incubation; (e) *Candida glabrata* 24 h of incubation; (f) *C. glabrata* 48 h of incubation.

*C. albicans* is a human opportunist pathogen that can grow as yeast, pseudohyphae, or true hyphae *in vitro* and *in vivo*, depending on environmental conditions. The hyphae are the vegetative form of filamentous fungi, which possess a thread-like structure. Pseudohyphae are chains of newly divided cells of unicellular fungi. The main difference between hyphae and pseudohyphae is their formation. *C. albicans* is a diploid yeast-like fungus. *C. albicans* cultures form large thick-walled chlamydoconidia at the ends of true hyphae or along the pseudohyphae. Structures similar to chlamydoconidia, but with thinner walls, are found in *C. tropicalis*. Blastoconidia, which are yeast cells themselves, are structures for asexual reproduction in all yeast-like fungi. They occur during budding and have the form of small round cells, which elongate to form pseudohyphae. Figure-2 shows preparations of the fungi *C. krusei* and *C. guilliermondii*, and pseudohyphae of various shapes are clearly visible. *C. glabrata* is a haploid organism and is not able to form mycelial structures.

### Effect of farnesol on the formation of *Candida* biofilms

*C. albicans* biofilms formed in the first, control well with an average value of OD of 0.485. This value indicates that strain ATCC 10231 is a strong producer of biofilms. *C. krusei* and *C. guilliermondii* are average producers of biofilms, while *C. glabrata* is a weak producer of biofilms (Table-2).

**Table-2:** Average results of three densitometric studies.

Farnesol concentration	1	2	3	4	5	6	7	8	9	10	11	12
											
	Controlno farnesol	200 μM	100 μM	50 μM	25 μM	12.5 μM	6.3 μM	3.1 μM	1.6 μM	0.8 μM	0.4 μM	0.2 μM
*Candida albicans* ATCC 10231	0.485± 0.013	0.190±0.008	0.215±0.006	0.234±0.011	0.226±0.009	0.233±0.010	0.249±0.004	0.302±0.007	0.368±0.007	0.407±0.009	0.439±0.011	0.473±0.012
*Candida krusei*	0.355± 0.010	0.097±0.002	0.121±0.007	0.144±0.008	0.166±0.008	0.172±0.006	0.184±0.011	0.207±0.008	0.220±0.007	0.279±0.012	0.293±0.010	0.348±0.010
*Candida guilliermondii*	0.305± 0.006	0.103±0.003	0.113±0.004	0.140±0.004	0.174±0.007	0.185±0.009	0.234±0.014	0.252±0.012	0.262±0.009	0.279±0.010	0.293±0.014	0.298±0.008
*Candida glabrata*	0.206± 0.005	0.095±0.002	0.105±0.004	0.128±0.003	0.147±0.008	0.181±0.006	0.200±0.004	0.204±0.008	0.206±0.007	0.205±0.007	0.211±0.010	0.209±0.011

The effect of different concentrations of farnesol on the yeast-like fungi biofilms

Farnesol in concentrations from 100 to 200 mM reduced the OD of *Candida* by more than 1 times and blocked the primary adhesion and fixation of *C. albicans* on the plastic surface, which is one of the main indicators of virulence of this pathogen. When a microorganism has weak attachment ability, it is quickly removed from the body. Farnesol in concentrations from 0.8 to 50 mM significantly reduced the OD *C. albicans, C. krusei*, and *C. guilliermondii*.

**Figure-3:**
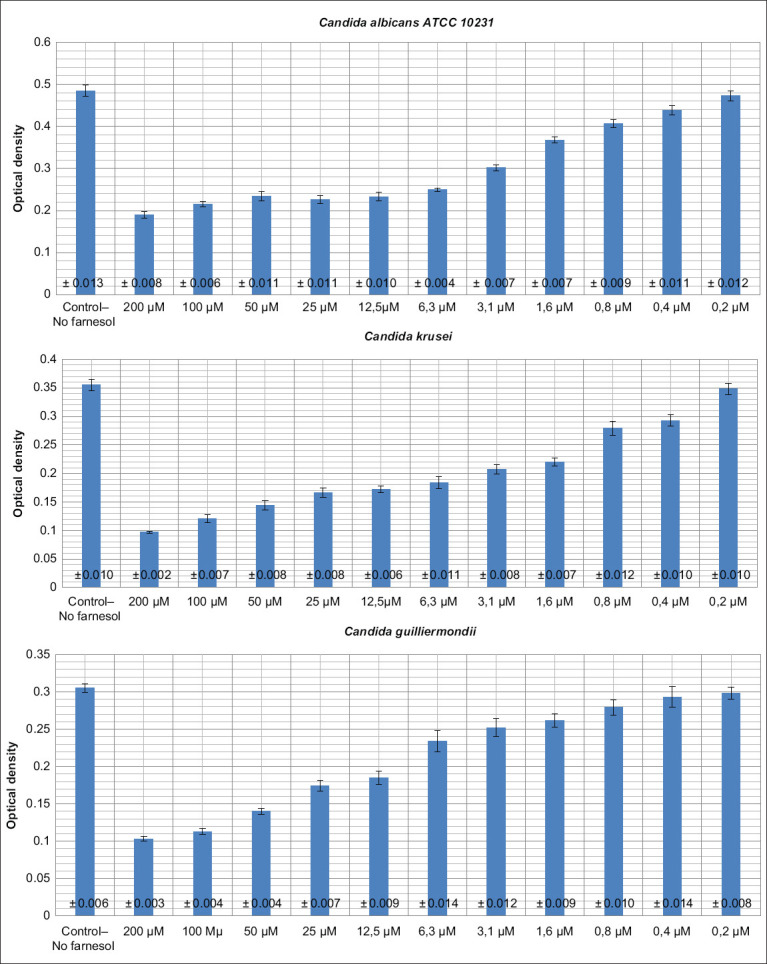
Graphical data on the dependence of farnesol concentration on the optical density of *Candida* biofilms.

*C. glabrata* turned out to be a weak biofilm producer. The morphofunctional stability of the biofilms of yeast-like fungi is provided by the yeast and hyphal forms. The process of germination ensures the development of the intercellular matrix. *C. glabrata* is not able to form hyphae and, therefore, cannot form strong biofilms compared to the other *Candida* species tested. Farnesol in concentrations of 12.5-50 mM significantly reduced the OD of *C. glabrata* compared to the control. Microdoses of farnesol at concentrations of 0.2-0.4 mM did not significantly affect the biofilms of any of the *Candida* species (Figure-3).

## Discussion

Yeast-like fungi of the genus *Candida* are found among the microflora of the mucous membranes of various animals and birds. *Candida* can be carried on the skin and mucous membranes for very long periods. In recent years, there has been a trend in the growth of candidiasis among people and animals. Fungi of the genus *Candida* are also very frequent participants in microbial associations [12-15].

In the most general and compressed terms, the development of *Candida* infection goes through a number of main pathogenetic stages: Adhesion of the pathogen to the surface of the mucous membrane or skin; invasive growth, with violation of the barrier functions of the skin and mucous membrane; and ­penetration of the pathogen into the blood, with generalization and the appearance of secondary foci in various tissues and organs.

Infectious diseases can produce complications due to the formation of microbial biofilms in the body. Many chronic fungal and bacterial diseases are associated with biofilms, since the biofilms adhere strongly to the cells of the human or animal body, and are less susceptible to antibiotic therapy than individual cells [16-21].

Multiple independent studies have proposed a positive correlation between *Candida* biofilm formation and an enhanced capacity for tissue invasion, damage, and virulence. The parasitic activities of different *Candida* species isolated in humans and animals are different. The most important pathogen is *C. albicans* followed by *C. tropicalis*. However, the significance of *C. krusei, C. guilliermondii*, and *C. glabrata* biofilms and their morphogenesis on pathogenicity has not been fully characterized. We investigated the interaction between the yeast forms, pseudohyphae, and hyphae of this fungus and the drug farnesol. We found that different farnesol concentrations reduced the OD of *Candida* species.

Farnesol is known to have a regulating and modifying effect on virulence factors such as colony color change, hyphae formation, and biofilm formation, of *Candida* spp., which determine their ability to infect a microorganism, with the development of local or systemic candidiasis. Farnesol inhibits the growth of fungi and also affects their biofilm formation and multidrug resistance.

## Conclusion

An understanding of these effects makes it possible to create synthetic drugs based on QS molecules that can control the intra- and inter-specific chemocommunication and virulence of fungi. The study of QS molecules that determine the life cycle of fungi, many of which are phytopathogenic or pathogenic to humans and animals, is of great importance for creating ­effective regulators of the fundamental life processes of fungal cells, including preparations with fungicidal activity. Understanding the molecular mechanisms of action of fungal QS molecules, and deciphering the structural and functional organization of the QS systems regulated by them, is also necessary for biotechnology based on the use of fungi for the production of biologically active substances, since the processes of fungal reproduction and development, as well as their biosynthetic activity, are under the direct control of these systems.

## Data Availability

Supplementary data can be available from the corresponding author.

## Authors’ Contributions

NS: Original idea for the study and carried out the design. NS and IP: Collected the samples, did data analysis and data cleaning. DB: Drafted the manuscript. The final draft manuscript was revised by all authors. All authors read and approved the final manuscript.
